# Parallel Paths, Uneven Progress: Health Care Provider and Patient Perspectives on Anti‐Amyloid Therapies for Early AD

**DOI:** 10.1002/alz70861_108349

**Published:** 2025-12-23

**Authors:** Vandana Gupta, Carole Drexel, Nathaniel A. Chin, Scott Kaiser, Dung Trinh, Nancy Lynn, Brooks Kenny, Monica Parsons, Sydnee Singer

**Affiliations:** ^1^ PlatformQ Health, Needham, MA USA; ^2^ Wisconsin AD Research Center, Madison, WI USA; ^3^ Pacific Neuroscience Institute, Santa Monica, CA USA; ^4^ Healthy Brain Clinic, Long Beach, CA USA; ^5^ BrightFocus Foundation, Clarksburg, MD USA; ^6^ National Infusion Center Association, Austin, TX USA; ^7^ Caregiver of Patient with AD, Los Angeles, CA USA

## Abstract

**Background:**

The management landscape for Alzheimer Disease (AD) is changing with the recent regulatory approvals of two anti‐ amyloid targeted agents for early AD in the United States, and additional agents are in the pipeline. We sought to characterize the current level of awareness, acceptance, and readiness among health care providers (HCPs) and patients/caregivers (P/Cs) to integrate these therapies into clinical care, using data collected during targeted educational initiatives.

**Methods:**

Two 60‐minute activities for P/Cs and two 60‐minute CME activities for HCPs were launched over the course of 2024. Pre‐ and post‐tests, polling, and baseline surveys assessed learner knowledge, confidence, and perceived barriers. Responses were analyzed for engagement, lessons learned, and continuing gaps. Chi‐square tests compared paired responses (P < 0.05; pre/post).

**Results:**

As of April 3, 2025, 944 HCPs (neurologists 14%, geriatricians 5%, primary care providers 25%) and 2,269 P/Cs engaged with the contents. Baseline HCP data highlighted deficiencies in their familiarity with diagnostic criteria (48% correct), evidence on anti‐amyloid therapy in the treatment of early AD (43% correct), and strategies for evaluating patients at risk for ARIA (50% correct). Participation led to significantly higher performance on the posttest (+23% increase on average), and enhanced confidence in engaging patients in treatment‐related discussions (22% pre vs 55% post). HCPs reported uncertainty around diagnosis, cost‐related constraints, and logistical challenges as the main barriers to utilizing newly approved anti‐amyloid therapies in clinical practice. Among P/Cs, 78% expressed interest in new therapies, but 56% were concerned about side effects, and many cited cost, limited access to specialists, and fear of insurance discrimination following an AD diagnosis as barriers to care. P/C learners submitted questions about blood‐based biomarker tests, trial eligibility, and strategies for engaging clinicians in the diagnostic process.

**Conclusions:**

HCPs continue to build up the knowledge and competence needed to diagnose early AD and address the complexities of integrating anti‐amyloid therapy in clinical practice. P/Cs are interested in new treatments but remain concerned about access, side effects, and stigma. These findings point to critical gaps in readiness and alignment that must be addressed to support the effective adoption of available therapies.

